# Host factors associated with respiratory particle emission and virus presence within respiratory particles: a systematic review

**DOI:** 10.3389/fmicb.2025.1652124

**Published:** 2025-10-15

**Authors:** Nils Horstink, Kirsten Lassing, Marjolein Knoester, Lucie C. Vermeulen, John W. A. Rossen, Andreas Voss, Mariëtte Lokate

**Affiliations:** ^1^Department of Medical Microbiology and Infection Prevention, University Medical Center Groningen, University of Groningen, Groningen, Netherlands; ^2^Department of Medical Microbiology, Radboud University Medical Center, Nijmegen, Netherlands; ^3^Centre for Infectious Disease Control, The National Institute for Public Health and the Environment (RIVM), Bilthoven, Netherlands; ^4^Laboratory of Medical Microbiology and Infectious Diseases, Isala Academy, Isala Hospital, Zwolle, Netherlands; ^5^Department of Pathology, University of Utah School of Medicine, Salt Lake City, UT, United States

**Keywords:** airborne transmission, infectious, respiratory particles, respiratory pathogen transmission, SARS-CoV-2, influenza, disease transmission, human

## Abstract

**Introduction:**

Understanding host factor-related mechanisms that drive variability in respiratory particle emission and virus presence in exhaled particles is essential to assess transmission risk and potentially identify individuals with elevated infectiousness.

**Methods:**

We conducted a systematic review of human observational studies examining associations between host factors and either respiratory particle emission or virus presence in exhaled particles. Searches in PubMed, EMBASE, and Web of Science covered studies up to September 2024. Risk of bias was assessed using STROBE-based criteria. Findings were synthesized narratively, grouped by host factor and outcome type.

**Results:**

Forty-four studies met inclusion criteria: 34 assessed host factors in relation to particle emission, and 11 examined viral presence in exhaled particles. Fine particle emission (<5 μm) was most consistently associated with older age (*n =* 16), physical exercise (*n =* 6), and active infection (*n =* 6). No consistent associations were found for sex (*n =* 21), body mass index (BMI; *n =* 10), or smoking (*n =* 6). Viral presence—mainly influenza and SARS-CoV-2—was more strongly associated with time since symptom onset (*n =* 8) and lower respiratory symptoms (*n =* 3), based largely on genomic detection. Associations with other factors, including upper respiratory symptoms (*n =* 6), swab viral load (*n =* 11), age (*n =* 6), sex (*n =* 6), and BMI (*n =* 2), were inconsistent or absent. Physical exercise was not evaluated in relation to viral presence.

**Discussion:**

Fine respiratory particles (<5 μm) were the predominant size fraction detected and often contained higher concentrations of viral RNA. Age, physical exercise, and active infection were consistently associated with increased emission of these particles. The presence of respiratory viruses in exhaled air was more strongly linked to infection-related factors such as early symptom onset and lower respiratory involvement. These patterns suggest distinct mechanisms contributing to airborne transmission. Interpretation was limited by methodological heterogeneity and predominant reliance on PCR. Still, consistent associations with host factors suggest their potential as indicators for transmission risk. As evidence focused mainly on influenza and SARS-CoV-2, generalizability is limited. Standardized methods and further research are needed to strengthen outbreak preparedness.

## 1 Introduction

Respiratory viruses continue to cause a substantial global burden of disease, resulting in millions of hospitalizations and considerable mortality each year (Organization WH, 2024; Sirota et al., 2025; Bender et al., 2024). Among the recognized transmission routes, airborne spread has gained increasing attention as a key contributor to the dissemination of these pathogens, especially in enclosed or poorly ventilated environments. The airborne route of transmission involves small virus-laden particles generated during routine expiratory activities, which can remain suspended in the air, travel over long distances, and deposit within the lower airways (Wang et al., 2021; Pöhlker et al., 2023).

**Figure 1:**
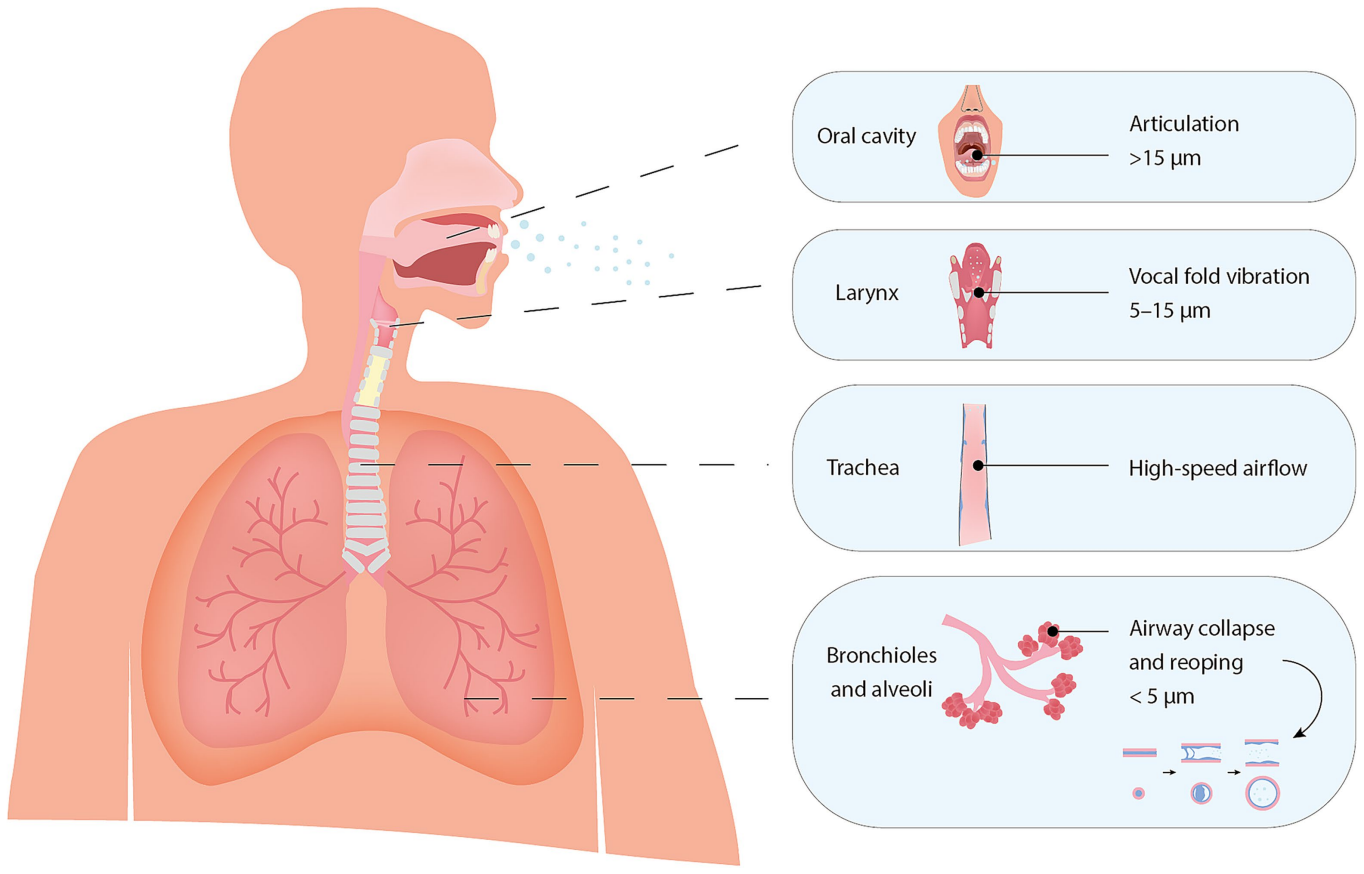
Anatomical sites of respiratory particle generation and associated particle size ranges at the moment of generation. The illustration highlights hypothesized generation sites based on Pöhlker et al. (2023) and Pan et al. (2023), with associated particle size ranges adapted from Bagheri et al. (2023), reflecting their estimated diameters at the point of generation.

However, respiratory particle generation is not a single, uniform process, but rather a complex and dynamic one that can be modulated by various physiological factors, such as airway structure, airflow dynamics, surface tension, and fluid rheology (Pöhlker et al., 2023; Bagheri et al., 2023; Johnson and Morawska, 2009; Almstrand et al., 2010; Roth et al., 2023; Edwards et al., 2004). In addition, different respiratory activities engage distinct regions of the airway, resulting in varied particle formation mechanisms and size distributions (Wang et al., 2021; Pöhlker et al., 2023; Kutter et al., 2018) (Figure 1). Fine respiratory particles (<5 μm) are primarily produced in the distal lung, where cyclic closure and reopening of small airways disrupts the airway lining fluid in the bronchioles and alveoli. Variations in tidal volume and breathing rate can further modulate this mechanism (Pöhlker et al., 2023; Bagheri et al., 2023; Johnson and Morawska, 2009; Almstrand et al., 2010; Bake et al., 2017; Morawska et al., 2009; Tinglev et al., 2016; Oldham and Moss, 2019). In more proximal regions, such as the larynx and oral cavity, particle generation is mainly driven by shear-induced fragmentation and fluid-film rupture caused by vocal fold oscillation, oral articulation, and increased airflow, typically resulting in larger particles (Pöhlker et al., 2023; Bagheri et al., 2023). Expulsive events such as coughing and sneezing commonly intensify these mechanisms, generating even broader particle size distributions (Pöhlker et al., 2023; Morawska et al., 2009; Zayas et al., 2012; Xie et al., 2009).

Importantly, transmission risk also depends on whether these exhaled particles contain infectious virus. Active infection at sites of particle generation, as discussed above, may contribute to viral presence in exhaled particles, although this relationship remains unconfirmed. Virus-specific factors, such as cellular tropism and strain specificity, along with host-related factors including immune competence and infection severity, likely further modulate virus presence (Wang et al., 2021; Puhach et al., 2023; Flerlage et al., 2021).

Despite growing understanding of the physiological mechanisms underlying respiratory particle formation and infection kinetics, considerable inter-individual variability in respiratory virus transmission persists, and its underlying drivers remain poorly understood. Epidemiological findings indicate that a small proportion of individuals contribute disproportionately to secondary transmission, highlighting the need to investigate host-specific drivers of transmission risk (Guo et al., 2023; Wegehaupt et al., 2023). Demographic factors (e.g., age, sex, body mass index), lifestyle factors (e.g., smoking status, physical activity), and infection-related factors (e.g., symptom onset, disease severity) have been associated with differences in airway characteristics, mucus properties, and viral shedding profiles (Lee et al., 2017; Dixon and Peters, 2018; Molgat-Seon et al., 2018; Schneider et al., 2021; Hussain-Alkhateeb et al., 2021; Marshall et al., 2020; Zanin et al., 2016; Sun et al., 2024; Li and Tang, 2021; Roman et al., 2016; Rathnayake et al., 2023; LoMauro and Aliverti, 2018). Through these effects, they may shape both the volume of particles produced and the likelihood that those particles carry infectious virus.

This systematic review synthesizes current evidence on the associations between host-related factors and both respiratory particle emission and the detection of virus in exhaled respiratory particles. Understanding how host factors influence both the generation and infectiousness of airborne particles can provide valuable proxies for transmission risk and offer insights into underlying physiological processes. By identifying individual-level determinants of airborne transmission, these findings aim to support risk assessment and guide public health strategies in clinical, occupational, and community settings, especially in preparation for future respiratory virus outbreaks.

## 2 Methods

### 2.1 Study design

This systematic review was conducted in accordance with the Preferred Reporting Items for Systematic Reviews and Meta-Analyses (PRISMA) guidelines (Page et al., 2021).

### 2.2 Strategy and selection process

On February 23, 2024, a comprehensive search was performed across three databases: PubMed (via NCBI), EMBASE (via Elsevier), and Web of Science. To ensure the inclusion of more recent articles, an additional search was conducted on September 30, 2024.

The search strategy was developed in consultation with a librarian and structured using the PEO framework. It incorporated search terms related to aerosols as the study problem, respiratory activities or viruses as the exposure of interest, and emissions as the outcome of interest. The following search strategy was used: (aerosol OR droplet nuclei) AND (respirator* OR cough* OR sneez* OR speak* OR speech* OR breath* OR shout* OR SARS* OR COVID* OR corona* OR virus* OR influenza OR flu OR rhinovirus OR common cold OR RSV OR infect*) AND (expel* OR exhal* OR emiss* OR emit*). The strategy was further refined to include relevant Medical Subject Headings (MeSH) and Emtree terms specific to PubMed and EMBASE.

To refine the search and minimize the inclusion of irrelevant studies, three additional exclusion string blocks were applied: (1) “terms related to the dental field, medical procedures, drug delivery, and environmental aerosols” to exclude studies focused on these specific contexts, (2) “limitation to human studies,” excluding animal-based research, and (3) “exclusion of review papers.

The identified articles were deduplicated, and two authors (NH and KL) independently screened them for eligibility with the use of SR-Accelerator (Bond University) (Clark et al., 2020). Discrepancies in the title and abstract screening were discussed by the two authors to determine final inclusion. Any remaining disagreements were resolved through discussion. The following automation tools were used during the process: Deduplicator, Screenatron, and Disputatron (Forbes et al., 2024).

### 2.3 Eligibility criteria and exclusion criteria

Full-text articles written in English were included if they addressed: (1) the size and quantity of emissions from human subjects, (2) the presence of viral particles in emissions and (3) the correlation between emissions and host factors, such as demographic characteristics, lifestyle factors, and respiratory infection characteristics.

Studies were excluded if they focused on mitigation or leakage, toxicity, deposition, aerosol generation procedures, non-respiratory pathogens, animal studies, reviews, conference papers, opinion/editorial pieces, or publications in languages other than English.

### 2.4 Study categorization, data extraction and processing

To facilitate analysis, included studies were grouped into two main categories based on their primary outcome measure: (I) quantification of particles during respiratory emissions and (II) detection of viral particles in respiratory emissions. Categorization was determined through a detailed review of each study’s objectives and methodologies to ensure accurate classification.

For each included study, data were extracted on study design, sample size, participant characteristics (including age range, sex distribution, body mass index, and smoking status), type of virus investigated, respiratory activities assessed, particle size fractions analyzed, and sampling methods used. Data extraction was performed using Microsoft Excel (version 16.92).

Given the heterogeneity in study methodologies and reporting formats, a narrative synthesis approach was adopted. Associations between host factors and outcomes were categorized as positive, negative, null, or inconsistent, based on the reported direction and statistical significance. Where available, effect measures such as odds ratios, correlation coefficients, and *p*-values were recorded. No meta-analyses were performed due to variability in study designs and outcome measures.

The studies collectively examined a range of host factors, including age, sex, body mass index (BMI), smoking status, exercise intensity, infection status, symptom presence and severity, symptom onset timing, viral load in clinical swabs, and vaccination status, in relation to respiratory particle emissions, or presence of viral particles.

Data analysis and table generation were performed using R (version 4.3.2) and RStudio (version 2024.04.2 + 764), utilizing the Tidyverse (version 2.0.0) and gt (version 0.10.0) packages (Team RC, 2023; Wickham et al., 2019; Iannone et al., 2023).

### 2.5 Quality assessment

The quality of all included studies was assessed using the Strengthening the Reporting of Observational Studies in Epidemiology (STROBE) guidelines for cohort, case–control, and cross-sectional studies (von Elm et al., 2007). An overview of the assessment results is provided in Supplementary Table 3.

### 2.6 Terminology statement

Following the latest WHO consultation report (Organization GWH, 2024), this review acknowledges the continuous size distribution of respiratory particles but adopts the commonly used classification distinguishing fine particles (< 5 μm) from coarse particles (≥ 5 μm).

## 3 Results

**Figure 2:**
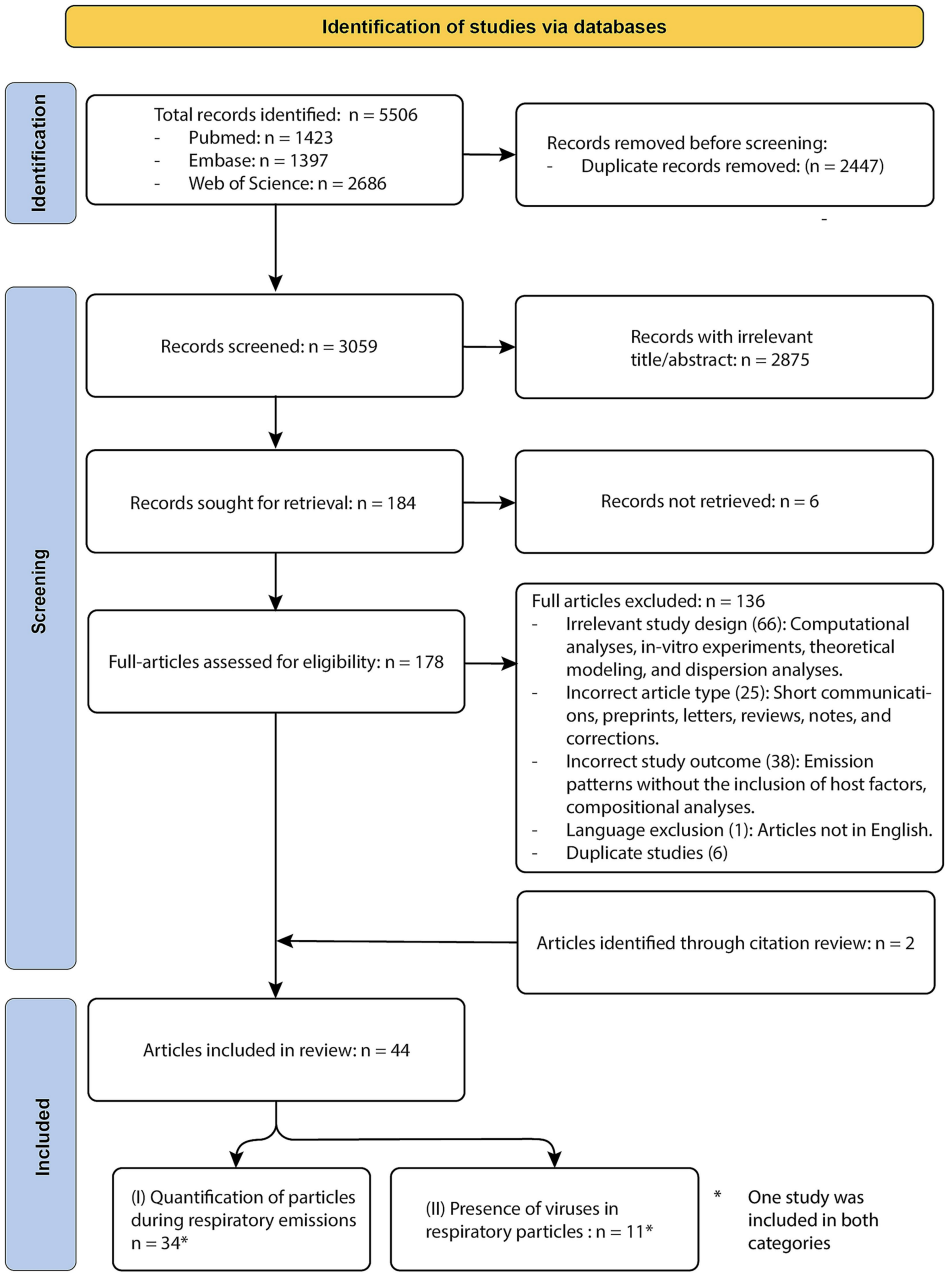
PRISMA flow diagram of study identification.

The database searches yielded a total of 5,506 articles. After deduplication, 3,059 unique citations remained and were screened by title and abstract. Of these, 2,875 were excluded for not meeting the inclusion criteria. An additional six articles were excluded due to retrieval issues, leaving 178 studies for full-text review. Following full-text screening, 136 articles were excluded due to ineligible study designs, outcomes, article types, language, or duplication. Two more studies were added through reference list screening, resulting in 44 studies meeting the eligibility criteria and included in this systematic review. Of these, host factor associations with respiratory particle emission were examined in 34 studies. An additional 11 studies assessed viral presence in exhaled particles in relation to host factors; all of these were limited to influenza virus or SARS-CoV-2. One study contributed to both outcome categories and is therefore included in both counts (Viklund et al., 2022). A detailed overview of the database-specific search terms and corresponding results is provided in Supplementary Tables 1, 2. Detailed reviewer comments and final decisions from both the title/abstract and full-text screening stages are provided in Supplementary File 1. A PRISMA flow diagram is provided in Figure 2.

### 3.1 Associations between host factors and respiratory particle emission

### 3.2 Study characteristics

Thirty-four studies investigating respiratory particle emission in relation to host factors were published between 2008 and 2024 (Table 1). A majority originated from Germany, the United States, and the United Kingdom. Most employed cross-sectional designs, with sample sizes ranging from small pilot cohorts to larger observational groups. Participant age across studies spanned from 2 to 87 years. A variety of respiratory activities were assessed, including breathing, speaking, singing, sustained phonation, and coughing. Sampling approaches differed across studies, resulting in the inclusion of a broad particle size range. Overall, the sampling window extended from 0.01 to 1,000 μm, although most studies focused on a narrower range between 0.3 and 10 μm. Inter-individual variability in respiratory particle emission was reported in 22 studies (Bagheri et al., 2023; Johnson and Morawska, 2009; Almstrand et al., 2010; Bake et al., 2017; Zayas et al., 2012; Viklund et al., 2022; Ahmed et al., 2022; Archer et al., 2022; Good et al., 2021; Gregson et al., 2021; Harrison et al., 2023; Lee et al., 2019; Lindsley et al., 2012; Mürbe et al., 2021; Mürbe et al., 2021; Mutsch et al., 2022; Pan et al., 2023; Rawat et al., 2023; Schuchmann et al., 2023; Schwarz et al., 2010; Schwarz et al., 2015; Gutmann et al., 2022). In contrast, six studies observed relatively stable intra-individual emission patterns across multiple sampling sessions (Bagheri et al., 2023; Almstrand et al., 2010; Bake et al., 2017; Rawat et al., 2023; Schwarz et al., 2010; Schwarz et al., 2015).

**Table 1:** Key characteristics of included studies assessing respiratory particle emission.

Author (Year)	Country	Study design	Number of participants (*n*)	Age range (years)	Size range (μm)	Measurement	Respiratory activities	Virus type (variants)	Investigated host factors	Reported correlations of host factors with respiratory particle emission	Quality
Schumm et al. (2024)	Germany	Cross-sectional	80	20–76	0.2–10	OPS	Breathing	-	Age, sex, BMI, smoking, exercise	Intensive exercise and age were positively associated with fine respiratory particle emission.	19/22
Moseley et al. (2024)	UK	Cross-sectional	33	29–63	0.54–20	APS	Breathing	-	Exercise	Exercise was positively associated with fine respiratory particle emission.	19/22
Schumm et al. (2023)	Germany	Cross-sectional	80	20–76	0.2–10	OPS	Breathing	-	age, sex, BMI, exercise	Age and intensive exercise were positively associated with fine respiratory particle emission. Among older individuals, females exhibited higher particle concentrations when at rest compared to males, with no differences in emission observed during exercise.	17/22
Bagheri et al. (2023)	Germany	Cross-sectional	132	5–80	0.1–1,000	CM	Various respiratory activities	-	Age, sex, BMI, smoking, exercise	Age was positively associated with fine respiratory particle emission. A non-significant positive trend was observed for smoking, though the analysis was limited by a small number of smokers in the study population.	19/22
Harrison et al. (2023)	UK	Prospective cohort	43	12–72	20–1,000	DA	Breathing, speaking, singing	-	Age, exercise	A slight increase in coarse particle emission (> 20 μm) was reported for exercise as compared to breathing, although with high variability.	19/22
Schuchmann et al. (2023)	Germany	Prospective cohort	250	2–17	0.145–10	OPS	Breathing	SARS-CoV-2 (Omicron)	Age, sex, BMI, smoking, infection, symptoms	Age and active infection were positively associated with fine respiratory particle emission.	19/22
Rawat et al. (2023)	USA	Prospective cohort	50	6–18+	0.54–20	APS	Vocalization (phonation)	-	Age	Age was positively associated with fine respiratory particle emission.	18/22
Pan et al. (2023)	China	Cross-sectional	12	20–39	0.3–20	APS	Breathing, speaking	-	Sex	Being male was positively associated with particle emissions during nasal breathing.	18/22
Varga et al. (2022)	USA	Cross-sectional	2	45–56	0.1–0.3	HS-LI	Breathing	-	exercise	High intensity exercise was negatively associated with submicron respiratory particle emission (~0.2 μm).	14/22
Orton et al. (2022)	UK	Cross-sectional	25	19–72	0.54–20	APS	Breathing, speaking	-	sex, exercise	Exercise was positively associated respiratory particle emission.	19/22
Mutsch et al. (2022)	Germany	Prospective cohort	16	18–40	0.2–10	OPS	Breathing	-	Sex, exercise	Exercise was positively associated respiratory particle emission.	16/22
Fleischer et al. (2022)	Germany	Cross-sectional	30	8–64	0.3–25	OPS	Breathing, speaking, singing, shouting	-	Age	Age was positively associated with respiratory particle emission.	18/22
Gutmann et al. (2022)	Germany	Prospective cohort	352	19–87	0.15–5	OPS	Breathing	SARS-CoV-2 (unspecified)	Age, sex, BMI, smoking, infection, symptoms, swab viral load	Age, active infection and higher viral load of clinical swabs were positively associated with fine particle emission.	21/22
Gutmann et al. (2022)	Germany	Prospective cohort	78	6–17	0.15–5	OPS	Breathing	SARS-CoV-2 (unspecified)	Age, sex, BMI, infection, swab viral load	Age, active infection and higher viral load of clinical swabs were positively associated with fine particle emission.	21/22
Archer et al. (2022)	UK	Cross-sectional	136	12–72	0.5–20	APS	Breathing, speaking, singing	-	Age, sex	Age was positively associated with respiratory particle emission.	18/22
Ahmed et al. (2022)	USA	Cross-sectional	40	20–60	0.53–20	APS	Vocalization (phonation)	-	Sex	Sex was associated with differences in respiratory particle emission at the two lowest vocalized notes, with males and females showing opposing patterns between the notes.	16/22
Viklund et al. (2022)	Sweden	Cross-sectional	36	23–67	0.4–5	CM	Breathing, coughing, combined respiratory activities	SARS-CoV-2 (unspecified)	Infection, swab viral load	Active infection was negatively associated with fine particle emission.	18/22
Sajgalik et al. (2021)	USA	Cross-sectional	8	20–63	0.3–10	OPS	Breathing	-	Exercise	Exercise was positively associated with increased particle emission, specifically at higher intensities.	18/22
Mürbe et al. (2021)	Germany	Cross-sectional	16	13–62	0.3–25	OPS	Speaking, singing	-	Age	Age was positively associated with respiratory particle emission, specifically while singing.	18/22
Good et al. (2021)	USA	Cross-sectional	63	12–61	0.25–33	OPS	Vocalization (speaking and singing combined)	-	Age, sex	Age was positively associated with particle emission. Males demonstrate a non-significant positive association.	19/22
Edwards et al. (2021)	USA	Cross-sectional	146	19–66	0.3–5	OPS	Breathing	-	Age, sex, BMI	The interaction between age and BMI showed a positive association with fine particle emission.	10/22
Mürbe et al. (2021)	Germany	Cross-sectional	8	22–62	0.3–25	OPS	Speaking singing	-	Sex	-	18/22
Gregson et al. (2021)	UK	Cross-sectional	25	22–57	0.523–20	APS	Vocalization (speaking and singing combined)	-	Sex	-	16/22
Kappelt et al. (2021)	Denmark	Cross-sectional	16	NA	0.3–10	OPS	Breathing, speaking	-	Sex	Females demonstrated a non-significant positive association in particle emission.	14/22
Asadi et al. (2019)	USA	Prospective cohort	48	18–45	0.5–20	APS	Speaking (phonation)	-	Age, sex, BMI	Age was positively associated with particle emission. BMI showed a non-significant positive association.	19/22
Lee et al. (2019)	Korea	Prospective cohort	10	22–33	0.01–10	OPS	Coughing	Acute respiratory infections (unspecified)	Sex, BMI, infection	Infection status was positively associated with particle emission.	20/22
Bake et al. (2017)	Sweden	Cross-sectional	126	41–66	0.41–4.55	OPS	Breathing	-	Age, sex, BMI, smoking	Age was positively associated with particle emission. BMI was negatively associated with particle emission in a multivariable model with age and the lung functions.	19/22
Schwarz et al. (2015)	Germany	Cross-sectional	67	20–74	0.1–10	CM	Breathing	-	Smoking	-	16/22
Zayas et al. (2012)	Canada	Cross-sectional	45	NA	0.1–900	OPS	Coughing	-	Age, sex	-	18/22
Lindsley et al. (2012)	USA	Prospective cohort	9	18–22	0.35–10	WPS	Coughing	Influenza virus (unspecified)	Infection	Active infection was positively associated with particle emission.	19/22
Schwarz et al. (2010)	Germany	Cross-sectional	21	21–63	0.1–10	CM	Breathing	-	Age	Age was positively associated with respiratory particle emission.	16/22
Almstrand et al. (2010)	Sweden	Cross-sectional	10	29–69	0.3–2.0	CM	Breathing	-	Age	-	15/22
Johnson et al. (2009)	Australia	Cross-sectional	17	19–60	0.3–20	APS	Breathing	-	Age	Age was positively associated with particle emission.	12/22
Hersen et al. (2008)	France	Case–control	78	6–66	0.07–10	ECI	Coughing	Acute respiratory infections (unspecified)	Sex, smoking, infection	Active infection was positively associated with particle emission.	15/22

USA, United States of America; UK, United Kingdom; CM, combined methods; WPS, wide-range particle spectrometer; OPS, optical particle sizer; APS, aerodynamic particle sizer; ECI, electrical cascade impactor; DA, deposition analysis; HS-LI, high-speed light imaging; BMI, body mass index.

### 3.3 Fine respiratory particle emission

Fine particle emissions were reported in most studies (*n =* 33), with seven studies focusing exclusively on this size fraction (Almstrand et al., 2010; Bake et al., 2017; Viklund et al., 2022; Gutmann et al., 2022; Gutmann et al., 2022; Edwards et al., 2021; Varga et al., 2022). Among the 26 studies that also included coarse particles, fine particles consistently represented the dominant mode of emission. A modal diameter of approximately 1 μm or smaller was reported in 21 studies (Bagheri et al., 2023; Johnson and Morawska, 2009; Almstrand et al., 2010; Zayas et al., 2012; Viklund et al., 2022; Ahmed et al., 2022; Archer et al., 2022; Good et al., 2021; Gregson et al., 2021; Mürbe et al., 2021; Mürbe et al., 2021; Mutsch et al., 2022; Rawat et al., 2023; Schuchmann et al., 2023; Schwarz et al., 2010; Schwarz et al., 2015; Gutmann et al., 2022; Asadi et al., 2019; Fleischer et al., 2022; Orton et al., 2022; Sajgalik et al., 2021; Schumm et al., 2023; Hersen et al., 2008). A slightly larger secondary mode, ranging from 1.3 to 2 μm, was identified in a subset of these studies (Archer et al., 2022; Good et al., 2021; Rawat et al., 2023; Orton et al., 2022). If coarse particles were detected, they were either infrequently observed or found at substantially lower concentrations (Archer et al., 2022; Gregson et al., 2021; Lindsley et al., 2012; Mürbe et al., 2021; Mürbe et al., 2021; Pan et al., 2023; Asadi et al., 2019; Fleischer et al., 2022; Sajgalik et al., 2021). Accordingly, the host-related associations described across studies were predominantly based on measurements within the fine particle fraction. Table 2 provides a summary of these findings, organized by host factor and reported direction of association.

**Table 2:** Summary of reported associations between host factors and fine respiratory particle emission.

Host factor	Association	Studies (*n*)	Population size (range)	Participant characteristics	Respiratory activities	References
Age	Positive association	16	16–288	2–87 years	Breathing, Vocalization	Bagheri et al. (2023), Johnson and Morawska, 2009), Bake et al. (2017), Archer et al. (2022), Good et al. (2021), Mürbe et al. (2021), Rawat et al. (2023), Schuchmann et al. (2023), Schwarz et al. (2010), Gutmann et al. (2022), Gutmann et al. (2022), Edwards et al. (2021), Asadi et al. (2019), Fleischer et al. (2022), Schumm et al. (2023), and Schumm et al. (2024)
	No association	2	10–45	29–69 years	Breathing, Coughing	Almstrand et al. (2010) and Zayas et al. (2012)
Sex	No association	18	8–288	48–59% male	Breathing, vocalization, coughing	Bagheri et al. (2023), Bake et al. (2017), Zayas et al. (2012), Archer et al. (2022), Good et al. (2021), Gregson et al. (2021), Lee et al. (2019), Mürbe et al. (2021), Mutsch et al. (2022), Schuchmann et al. (2023), Gutmann et al. (2022), Gutmann et al. (2022), Edwards et al. (2021), Asadi et al. (2019), Orton et al. (2022), Hersen et al. (2008), Schumm et al. (2024), and Kappelt et al. (2021)
	Context-specific associations	3	12–80	50–60% male	Breathing, vocalization	Ahmed et al. (2022), Pan et al. (2023), and Schumm et al. (2023)
BMI	No association	7	10–288	11.9–45 kg/m^2^	Breathing, vocalization, Coughing	Bagheri et al. (2023), Lee et al. (2019), Schuchmann et al. (2023), Gutmann et al. (2022), Gutmann et al. (2022), Schumm et al. (2023), and Schumm et al. (2024)
	Inconsistent associations	3	48–146	17–36 kg/m^2^	Breathing, vocalization	Bake et al. (2017), Edwards et al. (2021), and Asadi et al. (2019)
Exercise	Positive association	6	8–132	-	Breathing	Mutsch et al. (2022), Orton et al. (2022), Sajgalik et al. (2021), Schumm et al. (2023), Schumm et al. (2024), and Moseley et al. (2024)
	Negative association	1	2	-	Breathing	Varga et al. (2022)
Smoking	No association	6	67–288	5.3–55.2% smokers	Breathing, vocalization, Coughing	Bagheri et al. (2023), Bake et al. (2017), Schwarz et al. (2015), Gutmann et al. (2022), Hersen et al. (2008), and Schumm et al. (2024)
Tobacco exposure	No association	1	250	24% exposed	Breathing	Schuchmann et al. (2023)
Respiratory infection	Positive association	6	9–288	Various comparison groups	Breathing, Coughing	Lee et al. (2019), Lindsley et al. (2012), Schuchmann et al. (2023), Gutmann et al. (2022), Gutmann et al. (2022), and Hersen et al. (2008)
	Inconsistent association	1	36	Case vs. control	Breathing, coughing	Viklund et al. (2022)

Participant characteristics reflect the range or categories relevant to the host factors reported across studies within each association group. Some studies lacked participant-level data; sex was not reported in Edwards et al. (2021) and Mürbe et al. (2021), and BMI data were not in Lee et al. (2019) and Edwards et al. (2021). Sex-specific associations were observed during nasal breathing, low-frequency phonation, and at rest in elderly participants. Inconsistent associations with BMI reflect differences in the direction of association across studies. The smoker characteristic reflects the percentage of current smokers in the study population; smoking data were unavailable in Hersen et al. (2008) and Bake et al. (2017), while Gutmann et al. (2022) and Schumm et al. (2024) reported findings from subgroups only. The tobacco exposure characteristic refers to the percentage of pediatric participants exposed to household smoking. Infection-related comparisons included infected versus uninfected, symptomatic versus asymptomatic, and acute versus post-recovery phases. The inconsistent association for respiratory infection reflects variability across different respiratory activities, specifically breathing and coughing.

#### 3.3.1 Demographic factors

Age was the demographic factor most consistently associated with fine respiratory particle emission. Of the 18 studies evaluating this relationship, 16 reported increased emission rates with advancing age (Bagheri et al., 2023; Johnson and Morawska, 2009; Bake et al., 2017; Archer et al., 2022; Good et al., 2021; Mürbe et al., 2021; Rawat et al., 2023; Schuchmann et al., 2023; Schwarz et al., 2010; Gutmann et al., 2022; Gutmann et al., 2022; Edwards et al., 2021; Asadi et al., 2019; Fleischer et al., 2022; Schumm et al., 2023; Schumm et al., 2024). This trend was generally gradual, with higher emission observed across progressively older age groups. Associations were identified across a range of expiratory activities and population age spans, including pediatric and older adult participants. The two studies that did not report an association included smaller sample sizes and narrower age distributions (Almstrand et al., 2010; Zayas et al., 2012).

Sex was not associated with fine particle emission in most studies. Among the 21 studies assessing sex-related differences, 18 reported no significant variation between male and female participants (Bagheri et al., 2023; Bake et al., 2017; Zayas et al., 2012; Archer et al., 2022; Good et al., 2021; Gregson et al., 2021; Lee et al., 2019; Mürbe et al., 2021; Mutsch et al., 2022; Schuchmann et al., 2023; Gutmann et al., 2022; Gutmann et al., 2022; Edwards et al., 2021; Asadi et al., 2019; Orton et al., 2022; Hersen et al., 2008; Schumm et al., 2024; Kappelt et al., 2021). Most included balanced sex distributions and assessed multiple expiratory activities. Three studies reported context-specific differences under select conditions, such as nasal breathing, low-pitch phonation, or elevated emission in elderly females at rest; however, these observations were limited to isolated analyses and were not systematically evaluated across studies (Ahmed et al., 2022; Pan et al., 2023; Schumm et al., 2023).

As with sex, BMI was generally not associated with fine respiratory particle emission across studies. Ten studies assessed this relationship, most of which included participants with a broad range of BMI values. Seven studies reported no association (Bagheri et al., 2023; Lee et al., 2019; Schuchmann et al., 2023; Gutmann et al., 2022; Gutmann et al., 2022; Schumm et al., 2023; Schumm et al., 2024). The remaining studies described divergent patterns, including both positive and negative associations or interactions with other factors such as age, but these findings were limited to isolated analyses and were not replicated across the broader evidence base (Bake et al., 2017; Edwards et al., 2021; Asadi et al., 2019).

#### 3.3.2 Lifestyle factors

Among lifestyle-related factors, physical exercise demonstrated the most consistent association with fine respiratory particle emission. Six of seven studies reported increased fine particle emission during exercise, particularly at peak intensity, based on measurements taken before and during exertion on a cycle ergometer (Mutsch et al., 2022; Orton et al., 2022; Sajgalik et al., 2021; Schumm et al., 2023; Schumm et al., 2024; Moseley et al., 2024). One pilot study, which focused on particles in the lower submicron range, observed a reduction in emission during peak exercise (Varga et al., 2022).

In contrast, studies reported no associations between smoking and fine particle emission. Six studies assessed this relationship across various respiratory activities and populations with differing proportions of smokers; none identified significant differences between smokers and non-smokers (Bagheri et al., 2023; Bake et al., 2017; Schwarz et al., 2015; Gutmann et al., 2022; Hersen et al., 2008; Schumm et al., 2024). Similarly, one study evaluating secondhand smoke exposure in a pediatric population found no association (Schuchmann et al., 2023).

#### 3.3.3 Infection-related factors

The association between respiratory viral infections and fine particle emission was evaluated in seven studies. Of these, six studies observed increased emission during active infection, including investigations of SARS-CoV-2, influenza virus, and cases with confirmed viral respiratory infections lacking pathogen specification (Lee et al., 2019; Lindsley et al., 2012; Schuchmann et al., 2023; Gutmann et al., 2022; Gutmann et al., 2022; Hersen et al., 2008). Elevated emission was observed during both breathing and coughing in case–control comparisons, among symptomatic versus asymptomatic individuals, and during acute illness relative to post-recovery. In contrast to other findings, one study investigating SARS-CoV-2 reported reduced emission during breathing, while coughing showed no difference, indicating inconsistent associations across respiratory activities (Viklund et al., 2022).

Positive correlations between viral load in exhaled particles and clinical specimens were reported in two studies, though this relationship was not extensively investigated across the broader evidence base (Gutmann et al., 2022; Gutmann et al., 2022). Symptom-based comparisons were limited but did not yield statistically significant associations between fine particle emission and either upper or lower respiratory tract symptoms (Schuchmann et al., 2023; Gutmann et al., 2022).

#### 3.3.4 Coarse respiratory particle emission

Associations between host factors and coarse respiratory particle emission were only assessed in two studies, as the predominance of fine particles in other studies limited evaluation of coarse particle-specific effects (Bagheri et al., 2023; Harrison et al., 2023). These two studies investigated a broader particle size range, extending up to the millimeter scale, and were able to distinguish between coarse and fine fractions, or did not include the fine particle fraction at all. Despite the overall inclusion of a broad age range (5–80 years), no association was observed between age and coarse particle emission (Bagheri et al., 2023; Harrison et al., 2023). One study reported age-related differences restricted to particles smaller than 5–8 μm, suggesting that observed associations did not extend into the coarse size range (Bagheri et al., 2023). A modest increase in coarse particle emission was observed during vigorous exercise in one study; however, emissions in this size range were generally low and highly variable (Harrison et al., 2023).

### 3.4 Associations between host factors on the presence of respiratory viruses in exhaled particles

#### 3.4.1 Study characteristics

Eleven studies investigated associations between host factors and the presence of respiratory viruses in exhaled particles (Table 3). These studies, published between 2008 and 2024, were primarily conducted in the United States and employed cross-sectional or prospective cohort designs; one study was a single case report. Sample sizes ranged from individual cases to larger cohorts, with participant ages spanning 6 to 67 years.

**Table 3:** Key characteristics of studies evaluating viral presence in exhaled respiratory particles.

Author (Year)	Country	Study design	Number of participants (*n*)	Age range (years)	Size range (μm)	Respiratory activities	Measurement method	Virus (types/variants)	Viral detection method	Proportion of samples with detectable viral load (%)	Proportion of samples with viable virus (%)	Investigated host factors	Reported correlations of host factors with virus presence in respiratory particles	Quality
Jaumdally et al. (2024)	SA	Prospective cohort	44	27–71	0.65–10	combined respiratory activities	CI	SARS-CoV-2 (Beta, Delta, Omicron)	Culture	-	56.1	Age, sex, BMI, symptoms, symptom onset, swab viral load	Time since symptom onset was negatively associated with viability of virus in particles. Nasopharyngeal swab viral load showed a significant positive association, whereas saliva viral load demonstrated a non-significant positive association.	19/22
Lai et al. (2023)	USA	Prospective cohort	93	6–66	<5- > 5	combined respiratory activities	CI	SARS-CoV-2 (Ancestral/unspecified, Alpha, Delta, Omicron)	PCR	65.6	12.5	Age, sex, symptoms, symptom onset, swab viral load, vaccination status	Age was positively associated with particle viral load in both fine and coarse particles across all variants, but association was absent in Omicron-only analysis. Saliva and midturbinate swab viral loads were positively correlated with respiratory particle viral load in both fine and coarse particles across most variants, but positive associations were attenuated in Omicron cases. Upper and lower respiratory symptoms were significant predictors of viral load in fine particles across most variants, except for Omicron. Cough frequency showed a weak positive association with viral load in fine particles. Systemic symptoms were consistently associated with viral load in both fine and coarse particles.	15/22
Alsved et al. (2023)	Sweden	Case study	1	33–33	0.5–20	combined respiratory activities	CM	SARS-CoV-2 (Omicron)	PCR	100.0	-	Symptom onset, swab viral load	Time since symptom onset was negatively associated with presence of viral RNA in both fine and coarse respiratory particles.	21/22
Coleman et al. (2022)	Singapore	Cross-sectional	22	31–47	<5- > 5	combined respiratory activities	CI	SARS-CoV-2 (unspecified, Alpha, Beta, Kappa, Delta)	PCR	59.1	0.0	Age, sex, symptoms, illness onset, swab viral load	Time since illness onset was negatively associated with presence of viral RNA in both fine and coarse respiratory particles.	17/22
Viklund et al. (2022)	Sweden	Cross-sectional	36	23–67	0.4–5	combined respiratory activities	CM	SARS-CoV-2 (unspecified)	PCR	40.0	-	Age, sex, swab viral load	-	17/22
Chow et al. (2023)	Singapore	Cross-sectional	31	19–54	<5- > 5	combined respiratory activities	CI	Influenza virus A (H1N1, H3N2, H1N2) & Influenza virus B	PCR	41.9	29.0	Age, sex, symptom onset, swab viral load, vaccination status	Nasopharyngeal swab viral load showed a weak correlation with presence of viral RNA in fine respiratory particles.	19/22
de Mesquita et al. (2021)	USA	Prospective cohort	122	15–63	<5- > 5	combined respiratory activities	CI	Influenza virus A (H3N2)	PCR	68.0	-	Age, sex, infection, symptoms, swab viral load, symptom onset	Lower respiratory infection was positively associated with presence of viral RNA in fine and coarse respiratory particles. Nasopharyngeal swab viral load predicted RNA presence in fine particles only in nasally infected individuals, with a non-significant association observed in naturally infected cases. Lower respiratory and cough severity were positively associated with presence of viral RNA in fine respiratory particles in naturally infected cases. Time since symptom onset was negatively associated with presence of viral RNA in fine respiratory particles in naturally infected cases.	17/22
Yan et al. (2018)	USA	Prospective cohort	142	19–21	<5- > 5	combined respiratory activities	CI	Influenza virus A (pdmH1, H3) & Influenza virus B	PCR	76.1	23.9	Age, sex, BMI, smoking, symptoms, symptom onset, swab viral load, vaccination status	Male sex was associated with elevated viral load in fine particles. Cough frequency was a strong positive predictor of viral RNA presence in both fine and coarse particles. Time since symptom onset negatively correlated with the presence of viral RNA in fine particles, with weaker positive association observed in the coarse fraction. BMI was a significant positive predictor of viral RNA presence in fine respiratory particles.	20/22
Milton et al. (2013)	USA	Cross-sectional	37	18–54	<5- > 5	combined respiratory activities	CI	Influenza virus A (H1N1) & Influenza virus B	PCR	78.4	5.4	Smoking, symptom onset, swab viral load, vaccination status	Time since symptom onset negatively correlated with viral RNA presence in both fine and coarse particle fractions. Nasopharyngeal swab viral load showed a non-significant positive association with viral RNA presence in fine respiratory particles.	16/22
Lindsley et al. (2010)	USA	Cross-sectional	58	18–33	<1–4>	coughing	CC	Influenza virus A (H1N1)	PCR	84.2	5.3	Symptoms, swab viral load	Nasopharyngeal swab viral load was positive correlated with viral RNA presence in fine and coarse respiratory particles.	19/22
Fabian et al. (2008)	USA	Cross-sectional	12	14–61	0.3- > 5	combined respiratory activities	CM	Influenza virus A (H3) & Influenza virus B	PCR	33.3	-	Swab viral load	-	21/22

USA, United States of America; SA, South Africa; CM, combined methods; WPS, wide-range particle spectrometer; OPS, optical particle sizer; APS, aerodynamic particle sizer; ECI, electrical cascade impactor; DA, deposition analysis; HS-LI, high-speed light imaging; CI, cascade impactor; CC, cyclone collector; BMI, body mass index.

All studies involved outpatient populations with laboratory-confirmed Influenza virus or SARS-CoV-2 infection, though the viral variants and methods used to assess exhaled viral content varied. Most included multiple respiratory activities during sampling, precluding assessment of activity-specific associations with viral emission. An exception was one study that investigated associations during coughing alone (Lindsley et al., 2010). Particle size was commonly stratified into fine and coarse fractions using a single cut-off, although some studies applied multiple filter thresholds or combined physical sampling with optical sizing to enhance resolution. Viral presence was predominantly assessed using reverse transcription polymerase chain reaction (RT-PCR) to quantify viral RNA (hereafter referred to as viral load). Most infected individuals exhibited detectable viral load in exhaled particles, with consistently higher loads in fine compared to coarse fractions. Substantial inter-individual variability was reported in nine studies (Viklund et al., 2022; Lindsley et al., 2010; Chow et al., 2023; Coleman et al., 2022; de Mesquita et al., 2021; Fabian et al., 2008; Jaumdally et al., 2024; Lai et al., 2023; Milton et al., 2013). Although viral viability was assessed in six studies, only one investigated its relationship with host factors (Jaumdally et al., 2024). In general, viable virus was detected at much lower rates than viral RNA.

The inclusion of various influenza virus types and SARS-CoV-2 variants did not reveal any consistent strain- or subtype-specific patterns. A subset of studies explicitly reported the absence of such differences in their findings (Coleman et al., 2022; Milton et al., 2013; Yan et al., 2018). One study, however, observed some variability in a sub-analysis of SARS-CoV-2 variants, particularly when Omicron was analyzed separately from pooled SARS-CoV-2 variants (Lai et al., 2023). Although this observation was limited to a single study, it is considered alongside other findings related to host factor associations discussed below. Table 4 summarizes the identified associations for each host factor, stratified by virus type (influenza or SARS-CoV-2).

**Table 4:** Summary of reported associations between host factors and viral presence in exhaled respiratory particles, stratified by virus type.

Host factor	Virus	Association	Studies (*n*)	Population size (range)	Participant characteristics	References
Age	Influenza virus	No association	3	31–142	15–63 years	Chow et al. (2023), de Mesquita et al. (2021), and Yan et al. (2018)
	SARS-CoV-2	No association	3	22–36	20–71 years	Viklund et al. (2022), Coleman et al. (2022), and Jaumdally et al. (2024)
	SARS-CoV-2	Positive association	1	93	6–66 years	Lai et al. (2023)
Sex	Influenza virus	No association	3	31–142	49–65% male	Chow et al. (2023), de Mesquita et al. (2021), and Yan et al. (2018)
	SARS-CoV-2	No association	4	22–93	31–86% male	Viklund et al. (2022), Coleman et al. (2022), Jaumdally et al. (2024), and Lai et al. (2023)
BMI	Influenza virus	Positive association	1	142	20.9–25.5 kg/m^2^	Yan et al. (2018)
	SARS-CoV-2	No association	1	44	Normal-obese	Jaumdally et al. (2024)
Smoking	Influenza virus	No association	2	30–142	15–24% smokers	Milton et al. (2013) and Yan et al. (2018)
Exercise	-	-	0	-	-	-
Time since symptom onset	Influenza virus	Negative association	3	37–142	0–5	de Mesquita et al. (2021), Milton et al. (2013), and Yan et al. (2018)
	Influenza virus	No association	1	31	1–3	Chow et al. (2023)
	SARS-CoV-2	Negative association	3	1–44	0–7 / ≤8 vs. >8	Coleman et al. (2022), Jaumdally et al. (2024), and Alsved et al. (2023)
	SARS-CoV-2	No association	1	93	1–13	Lai et al. (2023)
Upper respiratory symptoms	Influenza virus	No association	3	58–142	Headache, sore throat, upper respiratory symptoms	Lindsley et al. (2010), de Mesquita et al. (2021), and Yan et al. (2018)
	SARS-CoV-2	No association	2	22–44	Respiratory symptoms, rhinorrhea, anosmia	Coleman et al. (2022) and Jaumdally et al. (2024)
	SARS-CoV-2	Positive association	1	93	Upper respiratory symptoms (non-specified)	Lai et al. (2023)
Lower / systemic respiratory symptoms	Influenza virus	Positive association	1	122	Lower respiratory symptoms (non-specified)	de Mesquita et al. (2021)
	Influenza virus	No association	1	142	chest tightness, shortness of breath, and cough, malaise, headache, muscle/joint ache, and swollen lymph nodes	Yan et al. (2018)
	SARS-CoV-2	Positive association	1	93	Lower and systemic respiratory symptoms (non-specified)	Lai et al. (2023)
Viral load of clinical samples	Influenza virus	No association	2	12–142	Nasopharyngeal swab, nasal and throat swab	Fabian et al. (2008) and Yan et al. (2018)
	Influenza virus	Inconsistent associations	3	31–122	Nasopharyngeal swab	Chow et al. (2023), de Mesquita et al. (2021), and Milton et al. (2013)
	Influenza virus	Positive association	1	58	Nasopharyngeal swab	Lindsley et al. (2010)
	SARS-CoV-2	No association	3	1–36	Clinical swab, nasopharyngeal swab, saliva	Viklund et al. (2022), Coleman et al. (2022), and Alsved et al. (2023)
	SARS-CoV-2	Positive association	2	44–93	Mid-turbinate swab, saliva, Nasopharyngeal swab	Jaumdally et al. (2024) and Lai et al. (2023)

Participant characteristics reflect the range or categories relevant to the host factors reported across studies within each association group. A positive association with age was observed in a study pooling SARS-CoV-2 variants but was not found in a sub-analysis restricted to Omicron. BMI classifications reflect the ranges or categories reported across the included studies. Inconsistent associations for influenza viral load in clinical samples reflect weak and opposing correlations, primarily in relation to fine respiratory particles. Characteristics related to time since symptom onset, symptom classifications, and viral load from clinical swabs are included where reported and reflect study-specific definitions. Physical exercise was not evaluated in the included studies.

#### 3.4.2 Demographic and lifestyle factors

Demographic and lifestyle factors generally showed no significant associations with the presence of viruses in exhaled respiratory particles; however, some factors lack sufficient coverage across studies to make firm conclusions. Age and sex were each assessed in seven studies, most of which reported no significant association with viral load or virus viability within exhaled particles (Viklund et al., 2022; Chow et al., 2023; Coleman et al., 2022; de Mesquita et al., 2021; Jaumdally et al., 2024; Lai et al., 2023; Yan et al., 2018). Study populations were predominantly adults, although some also enrolled pediatric participants, and sex representation varied across cohorts. A single positive association between age and SARS-CoV-2 viral load was reported; however, this association did not persist in a sub-analysis limited to Omicron cases (Lai et al., 2023). Compared to age and sex, associations involving BMI and smoking status were examined less frequently. Findings for BMI were mixed: one study reported no association with SARS-CoV-2 viability within exhaled particles, while another study identified a positive association for influenza viral load (Jaumdally et al., 2024; Yan et al., 2018). Smoking status was assessed only in the context of influenza virus, with no significant associations observed (Milton et al., 2013; Yan et al., 2018). Physical exercise was not evaluated in relation to viral emission in any included studies.

#### 3.4.3 Infection-related factors

Among infection-related factors, the time since symptom onset demonstrated the clearest overall association with virus detectability in exhaled respiratory particles. Of the eight studies evaluating this factor, Six studies consistently reported decreasing particle viral load or viability as time since symptom onset increased (Coleman et al., 2022; de Mesquita et al., 2021; Jaumdally et al., 2024; Milton et al., 2013; Yan et al., 2018; Alsved et al., 2023). However, one of the two studies that did not observe this association assessed a broader post-symptom onset interval than the other studies investigating this factor (Chow et al., 2023; Lai et al., 2023).

Associations with respiratory symptoms varied by anatomical site. Among six studies assessing upper respiratory tract symptoms, most found no significant association of symptom presence with viral load in exhaled particles (Lindsley et al., 2010; Coleman et al., 2022; de Mesquita et al., 2021; Jaumdally et al., 2024; Yan et al., 2018). One exception reported an association between upper respiratory symptoms and SARS-CoV-2 viral load, observed exclusively in the fine particle fraction and not seen in the Omicron-specific analyses (Lai et al., 2023). In contrast, the presence of lower respiratory symptoms, evaluated in three studies, was more frequently associated with elevated viral load in exhaled particles (de Mesquita et al., 2021; Lai et al., 2023). One study that did not report a significant association involved participants with predominantly mild symptoms (Yan et al., 2018). Additionally, statistical significance was not observed in analyses specifically restricted to the Omicron variant (Lai et al., 2023).

Correlations between viral load in clinical specimens (e.g., nasopharyngeal swabs or saliva) and exhaled particle viral load were assessed in eleven studies, yielding mixed findings. Five studies reported no significant correlation (Viklund et al., 2022; Coleman et al., 2022; Fabian et al., 2008; Yan et al., 2018; Alsved et al., 2023). Among the remaining studies, most reported positive correlations between swab viral load and viral load in both fine and coarse particle fractions, although the strength of these associations varied (Lindsley et al., 2010; de Mesquita et al., 2021; Jaumdally et al., 2024; Lai et al., 2023). Higher correlation estimates were reported for the Omicron variant compared to pooled SARS-CoV-2 variants, specifically for saliva samples (Lai et al., 2023). Two studies on Influenza reported correlations limited to the fine particle size fraction; however, the direction of these correlations was opposite (Chow et al., 2023; Milton et al., 2013).

## 4 Discussion

This systematic review synthesizes evidence from 44 studies to elucidate if host determinants are associated with two critical aspects of airborne transmission: the generation of exhaled aerosol particles and the incorporation of infectious virus into those particles. Most host factor associations were reported for fine particles (<5 μm), reflecting both their abundance in exhaled respiratory particles and the methodological challenges inherent in assessing coarse particle fractions. Several host factors, including age, physical exercise, and active infection, emerged as consistent predictors of increased fine particle emission, whereas sex, BMI, and smoking yielded limited or inconsistent associations. Viral presence within these particles was most strongly linked to infection-related characteristics, particularly time since symptom onset and lower respiratory tract involvement, rather than demographic or lifestyle factors.

The host factor associations identified in this review may be explained by several underlying biophysical processes governing respiratory particle generation. As previously discussed, the cyclic closure and reopening of small airways in the distal lung is a primary mechanism contributing to the formation of fine particles (Figure 1). This process may vary across the lifespan. In younger individuals, ongoing alveolar development into early adulthood may limit the surface area available for particle formation, contributing to lower emission levels. In contrast, older adults exhibit reduced elastic recoil and increased airway collapsibility, which may enhance airway reopening and shear forces, thereby promoting increased fine particle generation (Roman et al., 2016; Thomas et al., 2019; Martins Rodrigues et al., 2021; Nikolić et al., 2018). Age-related changes in mucus composition may further influence fluid rheology and reduce surface tension thresholds, facilitating droplet fragmentation (Edwards et al., 2004; Hussain-Alkhateeb et al., 2021). Physical exercise also acts along this pathway by increasing tidal volume and airflow velocities, thereby amplifying shear forces and the frequency of airway reopening events. These mechanical stresses promote fragmentation of the airway surface liquid and enhance fine particle emission (Johnson and Morawska, 2009; Morawska et al., 2009; Archer et al., 2022; Harrison et al., 2023). However, systemic dehydration during intense exercise may reduce airway hydration, potentially limiting submicron particle production despite elevated ventilation (Marshall et al., 2020; Varga et al., 2022). A respiratory infection may similarly enhance particle output by increasing mucus production and airway surface liquid, or indirectly through elevated respiratory rates associated with shortness of breath (Li and Tang, 2021; Weston and Frieman, 2019). Positive correlation reported between viral load in clinical swabs and the quantity of fine respiratory particles may reflect immune-mediated effects on airway physiology, although this hypothesis requires further validation. One contradictory finding was reported, suggesting that changes in viscosity may reduce particle emissions during infection. However, the study was limited by its small sample size as well as the duration and volume of sampling (Viklund et al., 2022).

Host factors such as sex, BMI, and smoking were not consistently associated with fine particle emission. While it is known that these factors can influence respiratory tract physiology, their effects on particle generation may be indirect or context dependent. For example, the lack of sex differences may reflect comparable alveolar structure and lung function across sexes in adulthood (Molgat-Seon et al., 2018; LoMauro and Aliverti, 2018). Occasional findings, such as increased emission during nasal breathing or low-frequency phonation, suggest a minor role for proximal production mechanisms, although supporting evidence remains limited. Variable associations with BMI may relate to comorbid conditions such as asthma or COPD, which can affect airway reopening dynamics (Sun et al., 2024). Similarly, although smoking is known to alter the bronchial mucus barrier and increase mucus production, it does not appear to enhance fine particle formation (Rathnayake et al., 2023). One possible explanation, also noted in the context of infection (Viklund et al., 2022), is that increased viscosity may limit the fragmentation of the airway lining fluid, thereby reducing fine particle emission and potentially masking any effect of smoking, although this remains speculative (Edwards et al., 2004).

By contrast, viral loading of respiratory particles appears to depend more on infection biology than on the mechanics of particle generation. Findings regarding host factor associations were largely consistent across influenza virus and SARS-CoV-2, indicating similar patterns in viral load dynamics despite differences in virus type and variant. However, the lack of studies on other respiratory viruses constrains the generalizability of these observations. This limitation may relate to differences in tissue tropism, as seen with Omicron, which shows altered anatomical targeting compared to earlier variants and may contribute to virus-specific patterns of respiratory particle emission (Lai et al., 2023; Chatterjee et al., 2023).

Viral replication is reported to peak during the early phase of infection, likely contributing to a higher probability that exhaled particles contain infectious virus (Puhach et al., 2023; Chen et al., 2021). This aligns with consistent findings of a temporal decline in exhaled viral load as time since symptom onset increases. However, more severe infections can result in prolonged viral loads in the lower respiratory tract, potentially explaining the absence of a temporal decline in some studies (Puhach et al., 2023; Lim et al., 2021). The anatomical focus of infection appears to also influence the likelihood of detecting virus in exhaled particles. Specifically, associations between viral load in particles and lower respiratory tract symptoms were more frequently observed than with upper respiratory symptoms, which suggest independent mechanisms. This is consistent with studies reporting that especially involvement of the lower respiratory tract increases the likelihood of virus presence in exhaled particles (de Mesquita et al., 2021; Yan et al., 2018; Lindsley et al., 2016). Inconsistent correlations between swab-based viral load and exhaled viral particles underscore this anatomical disconnect. Notably, studies focusing specifically on coughing, which generates high-velocity shear-driven emissions, have demonstrated stronger correlations with upper airway viral load, suggesting that these clinical parameters may be more closely linked to proximal particle generation mechanisms (Lindsley et al., 2010; de Mesquita et al., 2021; Yan et al., 2018).

Demographic and lifestyle host factors did not consistently account for the presence of virus in exhaled particles. Although age was frequently associated with increased fine particle emission, it was less informative in identifying individuals likely to shed infectious virus. This may be attributed to the predominance of studies involving mildly symptomatic or outpatient populations, in whom minimal lower respiratory tract involvement may limit the generation of virus-laden particles from distal airways. However, a few studies did report positive associations for age and BMI, which may suggest potential links. Other physiological processes, particularly differences in immune response such as impaired immune clearance associated with advancing age or elevated BMI, could contribute to these associations (Flerlage et al., 2021; Schneider et al., 2021).

It is important to note that most reported associations between host factors and the presence of respiratory viruses are based on detection of genomic material rather than replication-competent virus. This detection shows that viral material can be present even in small particles, but infectious virus is less often recovered in the current evidence base. Therefore, RNA-based findings should be regarded as indicators of viral shedding rather than direct proof of infectiousness.

A limited number of studies assessed host factor associations with coarse particle emission. However, the findings suggest that the effects of age and physical exercise were weaker for coarse particles than for fine particles. The limited evidence likely reflects methodological constraints: sampling interfaces such as masks and tubing may reduce capture of larger particles due to impaction losses and flow restrictions, while dehydration and shrinkage may further bias size classification (Pöhlker et al., 2023; Bagheri et al., 2023; Edwards et al., 2004). Beyond particle size bias, other methodological constraints were common across studies. The majority were cross-sectional and conducted at a single time point, limiting the ability to capture dynamic changes in respiratory particle output or viral shedding over time. Few studies accounted for individual-level factors such as hydration status, which may influence particle generation (Gregson et al., 2021; Rawat et al., 2023; Oh et al., 2024). Heterogeneity in instrumentation and study protocols introduced variability across findings, making it difficult to isolate the absolute effect of host factors (Hu et al., 2024; Verreault et al., 2008). Symptom classification often relied on self-reported data, introducing potential misclassification.

Despite methodological variability, the inclusion of diverse study designs strengthens the overall conclusions of this review. Studies maintained internal consistency in sampling and analysis, allowing meaningful comparisons within cohorts. By evaluating both respiratory particle emission and viral presence, this review provides an integrated perspective on host-related determinants of airborne transmission. The findings suggest that certain host characteristics, particularly when considered in combination, may serve as practical indicators of infectiousness. Individuals with increased particle emission and a greater likelihood of virus presence, such as older adults in the early phase of infection or those with lower respiratory involvement, may benefit from targeted mitigation strategies. Future studies should examine the combined impact of host factors on viable viruses in respiratory particles, explore less-studied variables such as the effect of physical exercise, and include a broader range of respiratory pathogens. In parallel, investigating the underlying generation mechanisms that drive these associations may clarify causal pathways and strengthen mechanistic understanding. The adoption of standardized measurement approaches that include the coarse particle range, along with expanded clinical characterization in longitudinal studies, will be essential for improving data comparability and strengthening outbreak preparedness.

## 5 Conclusion

This systematic review highlights that host factors influence airborne transmission through two distinct mechanisms: airway biomechanics driving particle generation and infection biology determining viral presence in exhaled particles. Older age, physical exercise, and active respiratory infections were consistently associated with increased fine respiratory particle emission, whereas other variables, including sex, BMI, and smoking, showed no consistent associations or were insufficiently studied. Viral presence in respiratory particles was more closely linked to time since symptom onset and anatomical site rather than individual demographic or lifestyle factors. No single host factor explained both increased particle emission and viral presence, but combinations such as older age with lower respiratory involvement may better indicate transmission potential. Current evidence remains constrained by methodological heterogeneity, a predominant focus on SARS-CoV-2 and influenza, and reliance on genomic detection rather than viral viability. Closing these gaps will be essential not only for refining risk assessment and guiding targeted interventions, but also for strengthening outbreak preparedness and improving infection control strategies.

## Data Availability

The original contributions presented in the study are included in the article/Supplementary material, further inquiries can be directed to the corresponding author.
