# Supplementary material for: Host factors associated with respiratory particle emission and virus presence within respiratory particles: a systematic review
Source: Front Microbiol. 2025 Oct 15;16:1652124. doi: 10.3389/fmicb.2025.1652124 (PMC12568713; doi:10.3389/fmicb.2025.1652124)
Supplement: Supplementary file 2 [file Table_1.docx]

Supplementary Materials

Supplementary Table 1. Initial search strategy and results by database and conceptual block (23 February 2024).

| **Database** | **Block number** | **Search block** | **Operator** | **Block explanation** | **Number of results** |
| --- | --- | --- | --- | --- | --- |
| **PubMed** | #1 | ("Aerosols"[Mesh] OR "Respiratory Aerosols and Droplets"[Mesh] OR Aerosol*[tiab] OR “droplet nuclei”[tiab]) | AND | Terms indicative for aerosols | 70,557 |
|  | #2 | (Expel*[tiab] OR exhal*[tiab] OR emiss*[tiab] OR emit*[tiab]) | AND | Terms indicative for emission | 8,010 |
|  | #3 | (Respirat*[tiab] OR SARS*[tiab] OR COVID*[tiab] OR Corona*[tiab] OR Influenza[tiab] OR Flu[tiab] OR Rhinovirus[tiab] OR "Common cold"[tiab] OR RSV[tiab] OR virus*[tiab] OR infect*[tiab] OR cough*[tiab] OR sneez*[tiab] OR speak*[tiab] OR speech*[tiab] OR breath*[tiab] OR shout*[tiab]) | AND | Terms indicative for virus or respiratory activity | 1,798 |
|  | #4 | (dental[ti] OR ortho*[ti] OR “generation procedure*”[ti] OR “generating procedure*”[ti] OR “healthcare procedure*”[ti] OR surgery[ti] OR “medical procedure*”[ti] OR delivery[ti] OR climate[ti] OR greenhouse*[ti] OR wastewater*[ti] OR “black carbon”[ti] OR pollution[ti]) | NOT | Exclude terms related to dental, medical procedures, delivery or the environment | 1,593 |
|  | #5 | ("animals"[MeSH] NOT "humans"[MeSH]) | NOT | Exclude animal related papers, limit to human related papers | 1,490 |
|  | #6 | "Review"[Publication Type] | NOT | Exclude review papers | 1,352 |
|  | #7 | #1 AND #2 AND #3 NOT #4 NOT #5 NOT #6 |  | Final search block PubMed | 1,352 |
| **EMBASE** | #1 | ('respiratory droplets and aerosols'/exp OR (Aerosol* OR ‘droplet nuclei’):ab,ti,kw) | AND | Terms indicative for aerosols | 81,432 |
|  | #2 | (Expel* OR exhal* OR emiss* OR emit*):ab,ti,kw | AND | Terms indicative for emission | 11,625 |
|  | #3 | (Respirat* OR SARS* OR COVID* OR Corona* OR Influenza OR Flu OR Rhinovirus OR 'Common cold' OR RSV OR virus* OR infect* OR cough* OR sneez* OR speak* OR speech* OR breath* OR shout*):ab,ti,kw | AND | Terms indicative for virus or respiratory activity | 2,137 |
|  | #4 | (dental OR ortho* OR ‘generation procedure*’ OR ‘generating procedure*’ OR ‘healthcare procedure*’ OR surgery OR ‘medical procedure*’ OR delivery OR climate OR greenhouse* OR wastewater* OR ‘black carbon’ OR pollution):ti | NOT | Exclude terms related to dental, medical procedures, delivery or the environment | 1,867 |
|  | #5 | ('animal'/exp NOT 'human'/exp) | NOT | Exclude animal related papers, limit to human related papers | 1,738 |
|  | #6 | ('review'/it OR 'conference abstract'/it) | NOT | Exclude review or conference papers | 1,311 |
|  | #7 | #1 AND #2 AND #3 NOT #4 NOT #5 NOT #6 |  | Final search block EMBASE | 1,311 |
| **Web Of Science** | #1 | TS=(Aerosol* OR “droplet nuclei”) | AND | Terms indicative for aerosols | 159,696 |
|  | #2 | TS=(Expel* OR exhal* OR emiss* OR emit*) | AND | Terms indicative for emission | 35,992 |
|  | #3 | TS=(Respirat* OR SARS* OR COVID* OR Corona* OR Influenza OR Flu OR Rhinovirus OR "Common cold" OR RSV OR virus* OR infect* OR cough* OR sneez* OR speak* OR speech* OR breath* OR shout*) | AND | Terms indicative for virus or respiratory activity | 3,169 |
|  | #4 | TI=(dental OR ortho* OR “generation procedure*” OR “generating procedure*” OR “healthcare procedure*” OR surgery OR “medical procedure*” OR delivery OR climate OR greenhouse* OR wastewater* OR “black carbon” OR pollution) | NOT | Exclude terms related to dental, medical procedures, delivery or the environment | 2,775 |
|  | #5 | DT==("REVIEW") | NOT | Exclude reviews | 2,553 |
|  | #6 | #1 AND #2 AND #3 NOT #4 NOT #5 |  | Final search block WOS | 2,553 |

Search terms, Boolean operators, and number of results retrieved for each conceptual block during the initial database search conducted on 23 February 2024. The search was performed across PubMed, Embase, and Web of Science, using structured blocks (e.g., aerosols, interventions) with MeSH terms and free-text keywords combined using Boolean logic. The number of results returned from each block per database is reported.

Supplementary Table 2. Second search strategy and results by database and conceptual block (23 February–30 September 2024).

| **Database** | **Block number** | **Search block** | **Operator** | **Block explanation** | **Number of results** |
| --- | --- | --- | --- | --- | --- |
| **PubMed** | #1 | ("Aerosols"[Mesh] OR "Respiratory Aerosols and Droplets"[Mesh] OR Aerosol*[tiab] OR “droplet nuclei”[tiab]) | AND | Terms indicative for aerosols | 72,310 |
|  | #2 | (Expel*[tiab] OR exhal*[tiab] OR emiss*[tiab] OR emit*[tiab]) | AND | Terms indicative for emission | 8,418 |
|  | #3 | (Respirat*[tiab] OR SARS*[tiab] OR COVID*[tiab] OR Corona*[tiab] OR Influenza[tiab] OR Flu[tiab] OR Rhinovirus[tiab] OR "Common cold"[tiab] OR RSV[tiab] OR virus*[tiab] OR infect*[tiab] OR cough*[tiab] OR sneez*[tiab] OR speak*[tiab] OR speech*[tiab] OR breath*[tiab] OR shout*[tiab]) | AND | Terms indicative for virus or respiratory activity | 1,880 |
|  | #4 | (dental[ti] OR ortho*[ti] OR “generation procedure*”[ti] OR “generating procedure*”[ti] OR “healthcare procedure*”[ti] OR surgery[ti] OR “medical procedure*”[ti] OR delivery[ti] OR climate[ti] OR greenhouse*[ti] OR wastewater*[ti] OR “black carbon”[ti] OR pollution[ti]) | NOT | Exclude terms related to dental, medical procedures, delivery or the environment | 1,664 |
|  | #5 | ("animals"[MeSH] NOT "humans"[MeSH]) | NOT | Exclude animal related papers, limit to human related papers | 1,558 |
|  | #6 | "Review"[Publication Type] | NOT | Exclude review papers | 1,416 |
|  | #7 | **#1 AND #2 AND #3 NOT #4 NOT #5 NOT #6 (Filter: from 23-02-2024)** |  | **Final search block PubMed** | **71** |
| **EMBASE** | #1 | ('respiratory droplets and aerosols'/exp OR (Aerosol* OR ‘droplet nuclei’):ab,ti,kw) | AND | Terms indicative for aerosols | 83,496 |
|  | #2 | (Expel* OR exhal* OR emiss* OR emit*):ab,ti,kw | AND | Terms indicative for emission | 12,097 |
|  | #3 | (Respirat* OR SARS* OR COVID* OR Corona* OR Influenza OR Flu OR Rhinovirus OR 'Common cold' OR RSV OR virus* OR infect* OR cough* OR sneez* OR speak* OR speech* OR breath* OR shout*):ab,ti,kw | AND | Terms indicative for virus or respiratory activity | 2,252 |
|  | #4 | (dental OR ortho* OR ‘generation procedure*’ OR ‘generating procedure*’ OR ‘healthcare procedure*’ OR surgery OR ‘medical procedure*’ OR delivery OR climate OR greenhouse* OR wastewater* OR ‘black carbon’ OR pollution):ti | NOT | Exclude terms related to dental, medical procedures, delivery or the environment | 1,969 |
|  | #5 | ('animal'/exp NOT 'human'/exp) | NOT | Exclude animal related papers, limit to human related papers | 1,837 |
|  | #6 | ('review'/it OR 'conference abstract'/it) | NOT | Exclude review or conference papers | 1,386 |
|  | #7 | **#1 AND #2 AND #3 NOT #4 NOT #5 NOT #6 (Filter: from 23-02-2024)** |  | **Final search block EMBASE** | **86** |
| **Web Of Science** | #1 | TS=(Aerosol* OR “droplet nuclei”) | AND | Terms indicative for aerosols | 169,189 |
|  | #2 | TS=(Expel* OR exhal* OR emiss* OR emit*) | AND | Terms indicative for emission | 38,111 |
|  | #3 | TS=(Respirat* OR SARS* OR COVID* OR Corona* OR Influenza OR Flu OR Rhinovirus OR "Common cold" OR RSV OR virus* OR infect* OR cough* OR sneez* OR speak* OR speech* OR breath* OR shout*) | AND | Terms indicative for virus or respiratory activity | 3,441 |
|  | #4 | TI=(dental OR ortho* OR “generation procedure*” OR “generating procedure*” OR “healthcare procedure*” OR surgery OR “medical procedure*” OR delivery OR climate OR greenhouse* OR wastewater* OR “black carbon” OR pollution) | NOT | Exclude terms related to dental, medical procedures, delivery or the environment | 3,015 |
|  | #5 | DT==("REVIEW") | NOT | Exclude reviews | 2,778 |
|  | #6 | **#1 AND #2 AND #3 NOT #4 NOT #5 (Filter: from 23-02-2024)** |  | **Final search block WOS** | **133** |

Number of results retrieved for each conceptual block during the second database search, which covered literature published between 23 February and 30 September 2024. The search was conducted in PubMed, Embase, and Web of Science. The structure and content of the search blocks were similar to those used in the initial search (see Supplementary Table 1), using the same MeSH terms, keywords, and Boolean logic adapted to each database. Filters for publication date and other limits were applied as specified in the method.

Supplementary Table 3. STROBE checklist assessment for all included studies.

| Study | Item 1 | Item 2 | Item 3 | Item 4 | Item 5 | Item 6 | Item 7 | Item 8 | Item 9 | Item 10 | Item 11 | Item 12 | Item 13 | Item 14 | Item 15 | Item 16 | Item 17 | Item 18 | Item 19 | Item 20 | Item 21 | Item 22 | Total score |
| --- | --- | --- | --- | --- | --- | --- | --- | --- | --- | --- | --- | --- | --- | --- | --- | --- | --- | --- | --- | --- | --- | --- | --- |
| Ahmed 2022 | No | Yes | Yes | Yes | No | No | Yes | Yes | Yes | No | Yes | Yes | Yes | No | Yes | Yes | Yes | Yes | Yes | Yes | No | Yes | 16 |
| Almstrand 2010 | No | Yes | Yes | Yes | No | No | Yes | Yes | Yes | No | No | No | Yes | Yes | Yes | Yes | Yes | Yes | Yes | Yes | No | Yes | 15 |
| Alsved 2022 | Yes | Yes | Yes | Yes | Yes | Yes | Yes | Yes | Yes | No | No | No | Yes | Yes | Yes | Yes | No | Yes | Yes | Yes | Yes | Yes | 18 |
| Archer 2022 | No | Yes | Yes | Yes | No | Yes | Yes | Yes | Yes | No | Yes | Yes | Yes | Yes | Yes | Yes | No | Yes | Yes | Yes | Yes | Yes | 18 |
| Asadi 2019 | No | Yes | Yes | Yes | Yes | Yes | Yes | Yes | Yes | No | Yes | Yes | Yes | No | Yes | Yes | Yes | Yes | Yes | Yes | Yes | Yes | 19 |
| Bagheri 2023 | No | Yes | Yes | Yes | No | Yes | Yes | Yes | Yes | No | Yes | Yes | Yes | Yes | Yes | Yes | Yes | Yes | Yes | Yes | Yes | Yes | 19 |
| Bake 2017 | No | Yes | Yes | Yes | Yes | Yes | Yes | Yes | Yes | No | Yes | Yes | Yes | No | Yes | Yes | Yes | Yes | Yes | Yes | Yes | Yes | 19 |
| Chow 2023 | No | Yes | Yes | Yes | No | Yes | Yes | Yes | Yes | No | Yes | Yes | Yes | Yes | Yes | Yes | Yes | Yes | Yes | Yes | Yes | Yes | 19 |
| Coleman 2022 | No | Yes | Yes | Yes | No | No | Yes | Yes | No | No | Yes | Yes | Yes | Yes | Yes | Yes | Yes | Yes | Yes | Yes | Yes | Yes | 17 |
| Edwards 2021 | Yes | Yes | No | Yes | No | Yes | Yes | No | Yes | No | No | No | Yes | No | No | No | No | Yes | No | Yes | No | Yes | 10 |
| Fabian 2008 | Yes | Yes | Yes | Yes | Yes | Yes | Yes | Yes | Yes | No | Yes | Yes | Yes | Yes | Yes | Yes | Yes | Yes | Yes | Yes | Yes | Yes | 21 |
| Fleischer 2022 | No | Yes | Yes | Yes | No | No | Yes | Yes | Yes | No | Yes | Yes | Yes | Yes | Yes | Yes | Yes | Yes | Yes | Yes | Yes | Yes | 18 |
| Good 2021 | No | Yes | Yes | Yes | No | Yes | Yes | Yes | Yes | No | Yes | Yes | Yes | Yes | Yes | Yes | Yes | Yes | Yes | Yes | Yes | Yes | 19 |
| Gregson 2021 | No | Yes | Yes | Yes | No | Yes | Yes | Yes | Yes | No | Yes | Yes | No | No | Yes | Yes | Yes | Yes | Yes | Yes | No | Yes | 16 |
| Gutmann 2022 | Yes | Yes | Yes | Yes | Yes | Yes | Yes | Yes | Yes | No | Yes | Yes | Yes | Yes | Yes | Yes | Yes | Yes | Yes | Yes | Yes | Yes | 21 |
| Gutmann 2023 | Yes | Yes | Yes | Yes | Yes | Yes | Yes | Yes | Yes | No | Yes | Yes | Yes | Yes | Yes | Yes | Yes | Yes | Yes | Yes | Yes | Yes | 21 |
| Harrison 2023 | No | Yes | Yes | Yes | No | Yes | Yes | Yes | Yes | No | Yes | Yes | Yes | Yes | Yes | Yes | Yes | Yes | Yes | Yes | Yes | Yes | 19 |
| Hersen 2008 | Yes | Yes | Yes | Yes | No | No | Yes | Yes | Yes | No | Yes | Yes | Yes | No | Yes | Yes | Yes | Yes | No | Yes | No | No | 15 |
| Jaumdally 2024 | No | Yes | Yes | Yes | Yes | Yes | Yes | Yes | Yes | No | Yes | Yes | No | Yes | Yes | Yes | Yes | Yes | Yes | Yes | Yes | Yes | 19 |
| Johnson 2009 | No | Yes | No | Yes | No | Yes | Yes | Yes | Yes | No | No | No | Yes | No | Yes | Yes | Yes | Yes | No | Yes | No | Yes | 13 |
| Kappelt 2021 | No | Yes | Yes | Yes | No | No | Yes | Yes | Yes | No | Yes | Yes | No | No | Yes | No | Yes | Yes | Yes | Yes | No | Yes | 14 |
| Lai 2022 | No | Yes | Yes | Yes | No | No | Yes | Yes | No | No | Yes | Yes | Yes | No | Yes | Yes | Yes | Yes | Yes | Yes | No | Yes | 15 |
| Lee 2019 | No | Yes | Yes | Yes | Yes | Yes | Yes | Yes | Yes | Yes | Yes | Yes | Yes | No | Yes | Yes | Yes | Yes | Yes | Yes | Yes | Yes | 20 |
| Lindsley 2010 | No | Yes | Yes | Yes | Yes | No | Yes | Yes | Yes | No | No | No | Yes | Yes | Yes | Yes | Yes | Yes | Yes | Yes | Yes | Yes | 17 |
| Lindsley 2012 | No | Yes | Yes | Yes | Yes | Yes | Yes | Yes | Yes | No | No | Yes | Yes | Yes | Yes | Yes | Yes | Yes | Yes | Yes | Yes | Yes | 19 |
| Mesquita 2024 | No | Yes | Yes | Yes | No | Yes | Yes | Yes | Yes | No | Yes | Yes | Yes | No | Yes | Yes | Yes | Yes | Yes | Yes | No | Yes | 17 |
| Milton 2013 | No | Yes | Yes | Yes | Yes | No | Yes | Yes | No | No | Yes | Yes | No | Yes | Yes | Yes | Yes | Yes | Yes | Yes | no | Yes | 16 |
| Moseley 2024 | No | Yes | Yes | Yes | No | Yes | Yes | Yes | Yes | No | Yes | Yes | Yes | Yes | Yes | Yes | Yes | Yes | Yes | Yes | Yes | Yes | 19 |
| Murbe 2021 | No | Yes | No | Yes | Yes | Yes | Yes | Yes | Yes | No | Yes | Yes | Yes | No | Yes | Yes | Yes | Yes | Yes | Yes | Yes | Yes | 18 |
| Murbe 2021 (2) | No | Yes | Yes | Yes | No | Yes | Yes | Yes | Yes | No | Yes | Yes | Yes | No | Yes | Yes | Yes | Yes | Yes | Yes | Yes | Yes | 18 |
| Mutsch 2022 | No | Yes | Yes | Yes | No | Yes | Yes | Yes | Yes | No | Yes | Yes | Yes | No | Yes | Yes | Yes | Yes | Yes | Yes | No | No | 16 |
| Orton 2022 | No | Yes | Yes | Yes | No | Yes | Yes | Yes | Yes | No | Yes | Yes | Yes | Yes | Yes | Yes | Yes | Yes | Yes | Yes | Yes | Yes | 19 |
| Pan 2023 | No | Yes | Yes | Yes | No | No | Yes | Yes | Yes | No | Yes | Yes | Yes | Yes | Yes | Yes | Yes | Yes | Yes | Yes | Yes | Yes | 18 |
| Rawat 2023 | No | Yes | Yes | Yes | No | Yes | Yes | Yes | Yes | No | Yes | Yes | Yes | No | Yes | Yes | Yes | Yes | Yes | Yes | Yes | Yes | 18 |
| Sajgalik 2021 | No | Yes | Yes | Yes | No | No | Yes | Yes | Yes | No | Yes | Yes | Yes | Yes | Yes | Yes | Yes | Yes | Yes | Yes | Yes | Yes | 18 |
| Schuchmann 2023 | Yes | Yes | Yes | Yes | Yes | Yes | Yes | Yes | Yes | No | No | Yes | No | Yes | Yes | Yes | Yes | Yes | Yes | Yes | Yes | Yes | 19 |
| Schumm 2024 | No | Yes | Yes | Yes | No | Yes | Yes | Yes | Yes | No | Yes | Yes | Yes | Yes | Yes | Yes | Yes | Yes | Yes | Yes | Yes | Yes | 19 |
| Schumm 2023 | No | Yes | Yes | Yes | No | No | Yes | Yes | Yes | No | Yes | Yes | Yes | Yes | Yes | Yes | No | Yes | Yes | Yes | Yes | Yes | 17 |
| Schwarz 2010 | No | Yes | Yes | Yes | No | No | Yes | Yes | Yes | No | No | No | Yes | Yes | Yes | Yes | Yes | Yes | Yes | Yes | Yes | Yes | 16 |
| Schwarz 2015 | No | Yes | Yes | Yes | No | No | Yes | Yes | Yes | No | Yes | Yes | Yes | Yes | Yes | Yes | Yes | Yes | No | Yes | No | Yes | 16 |
| Varga 2022 | No | Yes | Yes | Yes | Yes | No | Yes | Yes | No | Yes | No | No | Yes | Yes | Yes | No | No | Yes | Yes | Yes | No | Yes | 14 |
| Viklund 2022 | No | Yes | Yes | Yes | Yes | Yes | Yes | Yes | Yes | No | Yes | Yes | No | Yes | Yes | Yes | Yes | Yes | Yes | Yes | No | Yes | 18 |
| Yan 2018 | No | Yes | Yes | Yes | Yes | Yes | Yes | Yes | Yes | No | Yes | Yes | Yes | Yes | Yes | Yes | Yes | Yes | Yes | Yes | Yes | Yes | 20 |
| Zayas 2012 | No | Yes | Yes | Yes | Yes | Yes | Yes | Yes | Yes | No | No | Yes | Yes | Yes | Yes | Yes | Yes | Yes | Yes | Yes | No | Yes | 18 |

Results of the STROBE checklist quality assessment for observational studies included in the review, using the STROBE checklist for cohort, case-control, and cross-sectional studies (combined), version 4. Each item (1–22) represents a standard reporting criterion. “Yes” indicates the study met the criterion; “No” indicates it did not. The final row (or column) shows the total number of criteria met per study.
